# 

**Published:** 1892-06

**Authors:** 


The eleventh International Medical Congress will be held in Rome, Italy, in September, 1893.

The Mississippi Valley Medical Association will hold its eighteenth annual session at Cincinnati, Ohio, Wednesday, Thursday, and Friday, October 12, 13, and 14, 1892. A large attendance and a valuable programme are expected. President, Chas. A. L. Reed, Cincinnati; secretary, E. S. McKee, Cincinnati.

The American Academy of Medicine will hold its seventeenth annual meeting at the Cadillac Hotel, Detroit, on Saturday, June 4th, and Monday, June 6, 1892, under the presidency of Dr. P. S. Connor, of Cincinnati. A number of papers will be read, and interesting discussions are promised.

The American Pharmaceutical Association will hold its fortieth annual meeting at the Profile House, White Mountain, N. H., commencing Thursday, July 14, 1892, at nine o'clock, A. M. An elaborate programme has been prepared, including visits and drives to the various points of interest in the vicinity of the meeting. The local secretary is H. M. Whitney, Lawrence, Mass., who should be addressed on all subjects pertaining to the meeting.

The Medical Society of the State of New York.—*Official Bulletin.*—The business committee of this society has been organized for the meeting of February, 1893, by the appointment of Dr. Seneca D. Powell, of No. 12 West Fortieth street, New York City, Chairman, and Drs. William Maddren, of Brooklyn, and John O. Roe, of Rochester, associates. The programme for the scientific work of the session is already well advanced in its preparation.

The American Electro-Therapeutic Association will hold its second annual meeting at the Academy of Medicine in New York City, October 4, 5, and 6, 1892, under the presidency of Dr. W. J. Morton. Dr. H. R. Bigelow is the Secretary.

The American Association of Obstetricians and Gynecologists will hold its fifth annual meeting at the Lindell Hotel, in St. Louis, on Tuesday, Wednesday, and Thursday, September 20, 21, 22, 1892, under the presidency of Dr. A. Vander Veer, of Albany.

The Medical Society of the County of Erie will hold its semi-annual meeting at the Y. M. C. A. Building, Mohawk, Pearl, and Genesee streets, on Tuesday, June 14, 1892, beginning at ten o'clock, A. M. Dr. Joseph Price, of Philadelphia, will read a paper entitled The Natural History of Pelvic Inflammation; Dr. Lewis S. McMurtry, of Louisville, Ky., will read a paper entitled The Relation of So-called Minor Gynecological Operations to Pelvic Disease ; Dr. Charles A. L. Reed, of Cincinnati, will read a paper on Methods of Early Diagnosis of Intra-Pelvic Disease ; and Dr. James F. W. Ross, of Toronto, will read a paper entitled Actual, Not Text-Book, Experience with Cases of Ectopic Gestation. Other papers have been promised, and a cordial invitation is extended to all physicians to attend the meeting.

### BOOKS RECEIVED.

A System of Gynecology, with 359 illustrations; based upon a translation from the French of Samuel Pozzi. Revised by Curtis M. Beebe, M. D., Chicago, Ill. New York: J. B. Flint & Co.

Report of the Commissioner of Education for the year 1888-89. Volume I. Containing: Part I., Chapters I. to X.—A General and Comparative Exhibit of Education in the United States and Foreign Countries. Part II., Chapters XI. to XXI.—Normal Schools, Manual Training, Courses of Study, etc. Washington: Government Printing Office. 1891.

Report of the Commissioner of Education for the year 1888-89. Volume II. Containing: Part III., Chapters XXII. to XXXV.—Detailed Statistics of Educational Systems and Institutions, with Comments and Discussions. Washington: Government Printing Office. 1891.

Transactions of the Medical Society of the State of North Carolina. Thirty-eighth annual session, held at Asheville, N. C., May 26, 27, and 28, 1891. Wilmington, N. C.: Jackson & Bell. 1892.

The Fourth International Prison Congress, St. Petersburg, Russia. By C. D. Randall, Official Delegate from the United States Bureau of Education. Circular of Information, No. 2, 1891. Washington, D. C.: Government Printing Office. 1891.

The Science and Art of Midwifery. By William Thompson Lusk, A. M., M. D., Professor of Obstetrics and Diseases of Women and Children in the Bellevue Hospital Medical College; Consulting Physician to the Maternity Hospital and to the Foundling Asylum; Visiting Physician to the Emergency Hospital; Gynecologist to the Bellevue and to the St. Vincent Hospitals; Honorary Fellow of the Edinburgh and London Obstetrical Societies; Corresponding Fellow of the Obstetrical Societies of Paris and Leipzig; Corresponding Fellow of the Paris Academy of Medicine, etc. New edition. Revised and enlarged, with numerous illustrations. New York: D. Appleton & Co. 1892.

Outlines of Zoology. By J. Arthur Thomson, M. A., F. R. S. E., Lecturer on Zoology in the School of Medicine, Edinbugh; joint-author of the Evolution of Sex; author of the Study of Animal Life. With thirty-two full-page illustrations. New York: D. Appleton & Co. 1892

Diseases of the Eye. A Hand-Book of Ophthalmic Practice for Students and Practitioners. By G. E. de Schweinitz, M. D., Professor of Diseases of the Eye in the Philadelphia Polyclinic; Lecturer on Medical Ophthalmoscopy in the University of Pennsylvania; Ophthalmic Surgeon to the Philadelphia Hospital, and to the Children's Hospital; Ophthalmologist to the Orthopedic Hospital and Infirmary for Nervous Diseases. With 216 illustrations and two chromo-lithograph plates. Philadelphia: W. B. Saunders, 913 Walnut street. 1892.

The Uses of Water in Modern Medicine. By Simon Baruch, M. D., Attending Physician to the Manhattan General Hospital and New York Juvenile Asylum; Consulting Physician to the Montefiore Home for Chronic Invalids; formerly Chairman of the Board of Health of South Carolina, etc., etc. Volume I. Detroit, Mich.: George S. Davis. 1892.

Diseases of the Nervous System. By J. A. Ormerod, M. D., Oxon., F. R. C. P., Lond., Medical Registrar and Demonstrator of Morbid Anatomy at St. Bartholomew's Hospital; Physician to the National Hospital for the Paralyzed and Epileptic, Queen Square, and to the City of London Hospital for Diseases of the Chest, Victoria Park. With numerous illustrations. Philadelphia: P. Blakiston, Son & Co., 1012 Walnut street. 1892.

Treatise on the Diseases of Women, for the use of students and practitioners. By Alexander J. C. Skene, M. D., Professor of Gynecology in the Long Island College Hospital, Brooklyn, N. Y.; formerly Professor of Gynecology in the New York Post-Graduate Medical School; Gynecologist to the Long Island College Hospital; President of the American Gynecological Society, 1887; Corresponding Member of the British, Boston, and Detroit Gynecological Societies, of the Royal Society of the Medical and Natural Societies of Brussels, and of the Leipzig Obstetrical Society; Fellow of the New York Academy of Medicine; Ex-President of the Medical Society of the County of Kings. Ex-President of the New York Obstetrical Society. Second edition, revised and enlarged, with 251 engravings and nine chromo-lithographs. New York: D. Appleton & Co. 1892.

Encyclopedia of Medicine and Surgery. By various writers, enlarged, upon a new system, which embodies the methods of treatment employed by eminent practitioners of medicine. Compiled under the direction of the publishers, and including the writings of Drs. John Abercrombie, E. C. Beale, C. E. Beevor, Sidney Coupland, Frederick F. Eve, G. P. Field, J. K. Fowler, Wm. Gay, W. B. Hayden, G. E. Hermance, Victor Horsley, Henry Juler, C. B. Keetley, and others. New York: J. B. Flint & Co. 1892.

Pye's Surgical Handicraft: A Manual of Surgical Manipulations, Minor Surgery, and other matters connected with the work of house-surgeons and surgical dressers. With 300 illustrations on wood. First American, from the third London, edition. Revised and edited by T. H. R. Crawle, F. R. C. S., Surgical Registrar to St. Mary's Hospital; and Surgical Tutor and Joint Lecturer on Practical Surgery in the Medical School. Complete in one volume. New York: E. B. Treat, 5 Cooper Union. 1892.

Diseases of the Nervous System. By Jerome K. Bauduy, M. D., LL.D., Professor of the Diseases of the Mind and Nervous System, and of Medical Jurisprudence, Missouri Medical College, St. Louis; late Physician-in-Chief to St. Vincent's Institution for the Insane; Corresponding Member of the New York Society of Neurology and Electrology; formerly Consulting Physician of the St. Louis County Lunatic

Asylum; Member of the New York Medico-Legal Society, etc. Second edition. Philadelphia: J. B. Lippincott Co. 1892.

The Electro-Therapeutics of Gynecology. By Austin H. Goelet, M. D., Fellow of the New York Academy of Medicine and of the New York Obstetrical Society; Vice-President of the American Electro-Therapeutic Association; Member of the Societé Francaise d'Electrotherapie; editor of the *Archives of Gynecology, Obstetrics, and Pediatrics.* Vols. I. and II. With illustrations. Physicians' Leisure Library. George S. Davis, Detroit, Mich. 1892.

Text-Book of the Eruptive and Continued Fevers. By John William Moore, B. A., M. D., M. Ch., Univ. Dubl.; Fellow and Registrar of the Royal College of Physicians of Ireland; Physician to the Meath Hospital, Dublin; Joint Professor of Practice of Medicine in the Schools of Surgery of the Royal College of Surgeons of Ireland; Consulting Physician to Cork-street Fever Hospital, Dublin, and to the Whitworth Hospital, Drumcondra; Ex-Scholar and Diplomat in State Medicine of Trinity College, Dublin. William Wood & Co., medical publishers, New York. 1892.

Eleventh Annual Report of the State Board of Health of Illinois, being for the year ended December 31, 1888. With an appendix, containing the Official Register of Physicians and Midwives, 1892. Springfield, Ill.: H. W. Rokker, State Printer and Binder. 1892.

Notice to Contributors.—We are glad to receive contributions from every one who knows anything of interest to the profession. Articles designed for publication in the Journal should be handed in before the first day of the month. The Editors are not responsible for the views or opinions of contributors. All communications should be addressed to the Managing Editor, 284 Franklin St., Buffalo, N. Y.

